# Capturing dynamics with Eiger, a fast-framing X-ray detector

**DOI:** 10.1107/S0909049512035972

**Published:** 2012-09-05

**Authors:** I. Johnson, A. Bergamaschi, J. Buitenhuis, R. Dinapoli, D. Greiffenberg, B. Henrich, T. Ikonen, G. Meier, A. Menzel, A. Mozzanica, V. Radicci, D. K. Satapathy, B. Schmitt, X. Shi

**Affiliations:** a Paul Scherrer Institut, 5232 Villigen PSI, Switzerland; b Soft Matter Group, ICS-3, Forschungszentrum Jülich, 52425 Jülich, Germany; c European Synchrotron Radiation Facility, 38043 Grenoble Cedex, France; d Department of Physics, Indian Institute of Technology Madras, Chennai 600036, India

**Keywords:** detector, single photon counting, X-ray diffraction, X-ray photon correlation spectroscopy

## Abstract

Eiger is the next-generation single-photon-counting pixel detector following the widely used Pilatus detector. Its smaller pixel size of 75 µm × 75 µm, higher frame rate of up to 22 kHz, and practically zero dead-time (∼4 µs) between exposures will further various measurement methods at synchrotron sources. In this article Eiger’s suitability for X-ray photon correlation spectroscopy (XPCS) is demonstrated. By exploiting its high frame rate, complementary small-angle X-ray scattering (SAXS) and XPCS data are collected in parallel to determine both the structure factor and collective diffusion coefficient of a nano-colloid suspension. For the first time, correlation times on the submillisecond time scale are accessible with a large-area pixel detector.

## Abstract 2

A high-frame-rate single-photon-counting pixel detector named Eiger and its suitability for X-ray photon correlation spectroscopy are described.

## 1. Introduction

Single-photon-counting area detectors have proven invaluable to many techniques at X-ray synchrotron sources (Henrich *et al.*, 2009 ▶). They have helped excel the fields of protein crystallography (Hilf & Dutzler, 2008 ▶), coherent diffractive imaging (Thibault *et al.*, 2008 ▶; Johnson *et al.*, 2008 ▶), small-angle X-ray scattering (Bunk *et al.*, 2009 ▶), time-resolved scattering studies (Ejdrup *et al.*, 2009 ▶; Westenhoff *et al.*, 2010 ▶), powder diffraction (Bergamaschi *et al.*, 2010 ▶) and X-ray photon correlation spectroscopy (Johnson *et al.*, 2009 ▶; Westermeier *et al.*, 2009 ▶). These fields all benefit from the precise measurement of the scattered X-ray intensity that is achieved with the combination of the sensitivity, linearity, low to non-existing readout noise, and the high dynamic range of large-area single-photon-counting detectors.

## 2. Detector

Eiger (Dinapoli *et al.*, 2011 ▶) is a new X-ray pixel detector that is similar in nature to the widely utilized and successful Pilatus detector (Broennimann *et al.*, 2006 ▶). It is composed of a silicon sensor, complementary metal-oxide-semiconductor (CMOS) readout chip, and readout electronics. X-rays deposit their energy in a pixelated silicon sensor that is typically 320 µm thick. Indium bump-bonds connect each sensor pixel to a pixel cell in the readout chip. Charge from the sensor is amplified and shaped, then compared with a threshold level by a discriminator which increments a 12-bit counter in the pixel cell. The counter values directly record the number of incident photons above threshold, *i.e* measure the intensity distribution across the sensor. Variations in the response across the chip are reduced by adjusting 6 in pixel trim-bits so that they effectively correct for individual pixel offsets to the global threshold. After the trim-bits have been tuned, the threshold dispersion across the chip is about 70 eV. A single chip is 19.3 mm × 20.1 mm and contains an array of 256 × 256 pixels on a 75 µm-pitch grid.

One set of counter values can be stored locally in the pixel cells of the Eiger readout chip, freeing the counters for the succeeding acquisition. Only ∼4 µs are needed between frames to stop the current exposure, store the counter values, reset and start the next exposure. The stored values are serialized in the periphery of the chip and read out on 32 pads with a double-data-rate (DDR) bus frequency of 100 MHz. Correspondingly, frame rates of 8 kHz, 12 kHz or 22 kHz can be achieved when 12-, 8- or 4-bit of the pixel counters are transferred.

**Figure 1:**
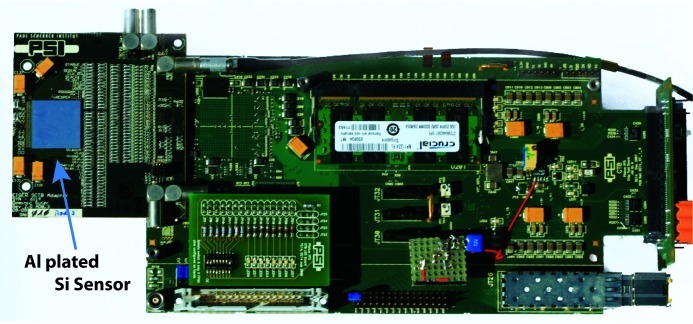
Photograph of an Eiger single-chip test system. The silver square on the left-hand side is the aluminium surface of the 2 cm × 2 cm silicon sensor, under which an Eiger readout chip resides. The overall length of the system is about 27 cm.

Data from the chip are buffered directly on the readout board in a standard 2 GB DDR2 memory module. Thus, a large series of images can be captured at the highest frame rate and stored before the transfer bottleneck to a computer over standard ethernet. A 65 kpixel single-chip system is shown in Fig. 1 ▶. It is a fully operational prototype for the 40 mm × 80 mm, 8-chip, 500 kpixel module system currently under development. Large-area multimillion-pixel detector systems will be constructed by tiling modules. Features of the single-chip test system will be preserved by the parallelization in larger multimodule systems and extended with data transfer over two 10 gigabit ethernet ports per module.

The major advantages over the Pilatus system are the smaller pixel size (a factor of five smaller in area) and higher frame rate (more than 50 times faster). We anticipate that these enhanced features will directly benefit several fields at X-ray synchrotron sources. In this paper we demonstrate Eiger’s applicability to X-ray photon correlation spectroscopy (XPCS). Since the detector is fully capable of measuring the time evolution of an entire small-angle X-ray scattering (SAXS) distribution, the structure factor *S*(*q*) and the collective diffusion coefficient *D*(*q*) can be accessed simultaneously. Here *q* = (4π/λ)sin(2θ/2) denotes the momentum transfer, 2θ is the scattering angle, and λ is the wavelength.

## 3. Experimental set-up

**Figure 2:**
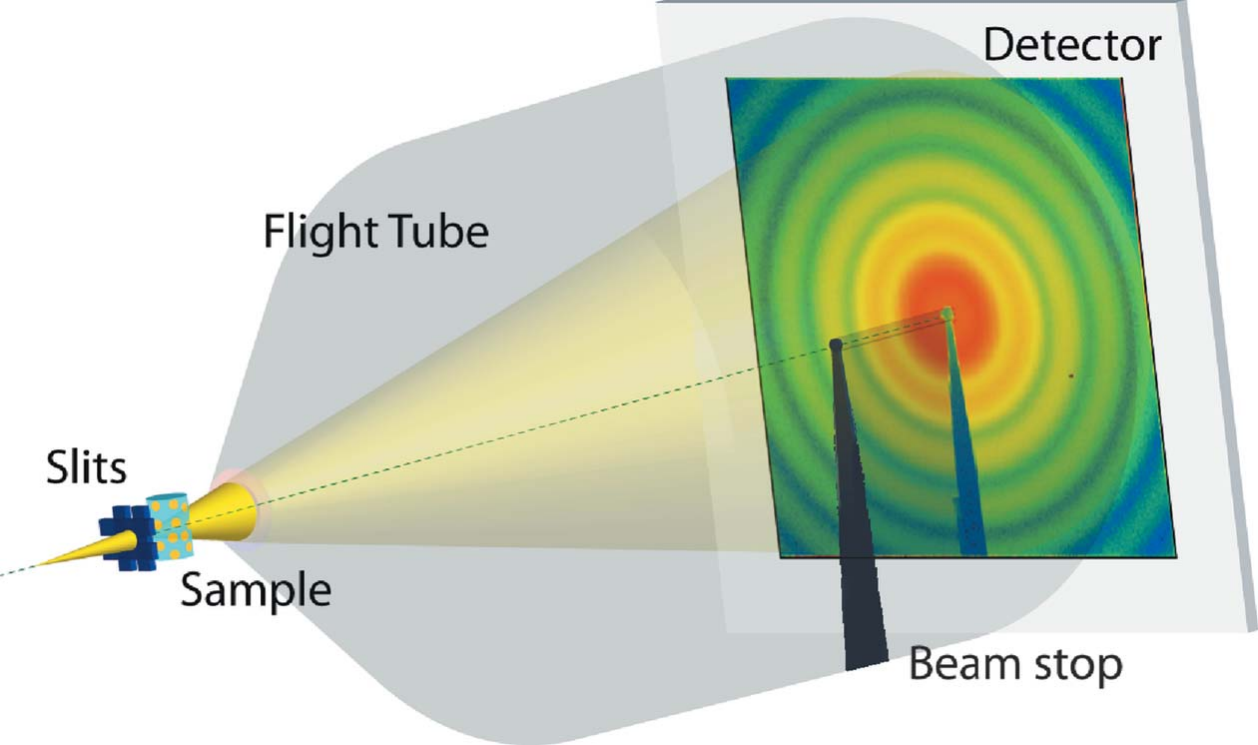
Experimental set-up.

The demonstration experiment was performed on the cSAXS beamline at the Swiss Light Source, Paul Scherrer Institut, Villigen, Switzerland. A portion of a slightly focused 8.7 keV (λ = 1.425 Å) beam from a double-crystal Si(111) monochromator was selected 34 m downstream from the undulator source [source size 200 µm × 20 µm full width at half-maximum (FWHM)]. Beam-defining slits, 20 µm × 20 µm, were located ∼65 cm upstream from the sample. The transmitted wave from the samples passed through a 2 m evacuated flight tube to the Eiger single-chip test system. A beam stop in the flight tube protected the detector from the direct beam and prevented scattering of the direct beam on the exit window of the flight tube. A schematic of the experimental set-up is shown in Fig. 2 ▶.

A sample with weakly screened electrostatic interactions was made from a 18% volume fraction suspension of 82 nm-radius silica colloids in dimethylformamide (DMF). Salt (LiCl) was added to the suspension to further induce screening of the electrostatic interaction. Along with these suspensions a dilute suspension to determine the form factor and a pure DMF solution to determine the background were investigated. All samples were measured in 1 mm-diameter quartz capillaries and prepared in a similar way to that described elsewhere (Gapinski *et al.*, 2009 ▶).

In 2.5 s, bursts of 50000 diffraction patterns were recorded with the Eiger single-chip system running at 20 kHz. The 50 µs period of each image was divided into a 45 µs exposure and 5 µs readout pause for temporally storing the current image on the chip and resetting the pixel counters. The system was operated in the 4-bit parallel-readout-exposure mode. The ∼1.5 GB image series was buffered in the memory of the test system and read out over the ethernet connection.

**Figure 3:**
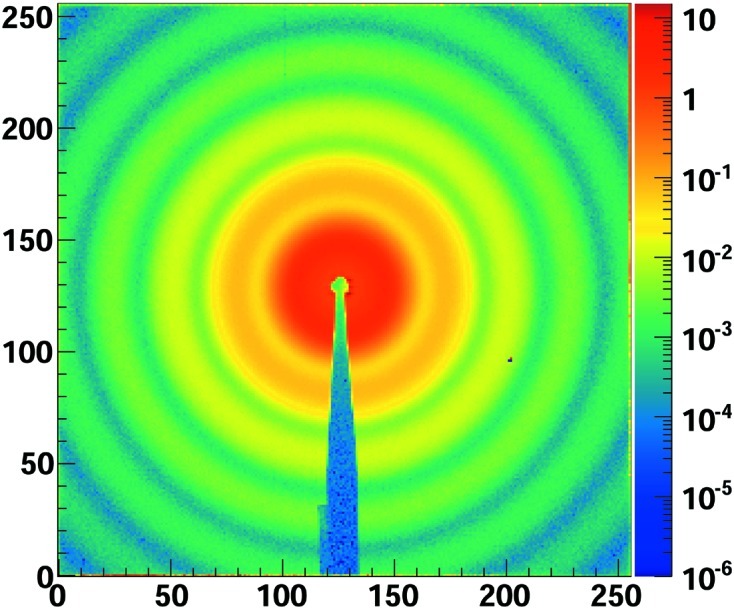
Average number of photons per frame for a 50000 image exposure series that was recorded in 2.5 s. The visibility of the outer rings in the plot, where pixels have one to a few detected X-rays during the complete exposure sequence, is a clear demonstration of the single-photon sensitivity of the detector.

**Figure 4:**
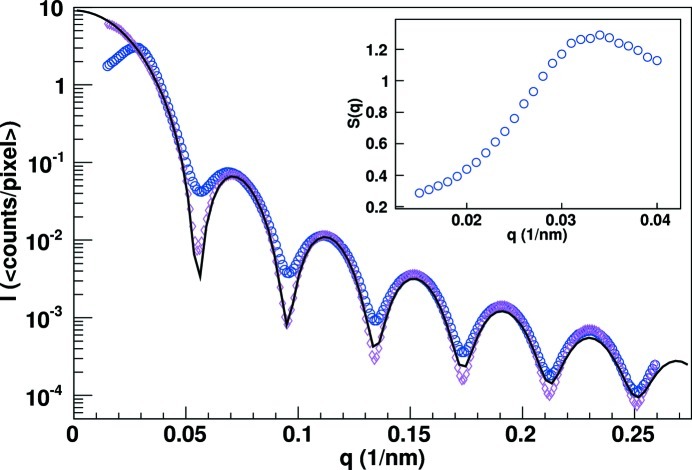
The average single 45 µs frame intensity *versus* *q* distribution of the dilute solution (pink diamonds), the weakly screened suspension (blue circles) and a polydisperse sphere form factor fit (black line). The structure factor for the weakly screened suspension is shown in the insert.

The average diffraction pattern from an exposure series is shown in Fig. 3 ▶. The tens of thousands of counts per pixel per second at low *q* are well within the dynamic range of the system, while the single-photon sensitivity is demonstrated by the few-photon response at larger *q* values. At a sample-to-detector distance of 2224 ± 40 mm the centered sensitive area of the single chip covered spatial frequencies up to *q* = 0.265 nm^−1^. The scattered intensity as a function of *q* was extracted by azimuthal averaging. The intensity distribution for the dilute and weakly screened (no salt) solutions are shown in the main plot of Fig. 4 ▶. The background measured with the pure DMF sample has been subtracted from these distributions. The particle size of 81 nm with a polydispersity of 3.5% has been determined by fitting the dilute solution data with a polydisperse sphere form factor model, also shown in the main plot of Fig. 4 ▶. Structure factors, one shown in the insert of Fig. 4 ▶, for the weakly screened and screened solutions were extracted by dividing the respective intensity distributions by that of the dilute sample, *i.e* the form factor.

## 4. XPCS with Eiger

XPCS is a technique for investigating equilibrium fluctuations in dynamical systems, such as suspensions and opaque soft materials. It is the X-ray analog of dynamic light scattering, a method that has proven to be a valuable tool for analyzing complex fluids. XPCS is frequently used to determine viscosity, hydrodynamic properties, glass transitions, or the profile distribution of particles in suspension or polymers in solution (Sikharulidze *et al.*, 2003 ▶; Falus *et al.*, 2005 ▶; Banchio *et al.*, 2006 ▶; Koga *et al.*, 2010 ▶).

Fundamentally, temporal fluctuations in the scatter intensity from a coherent monochromatic illumination on the specimen, the so-called speckle pattern, are measured. In particular, the intensity autocorrelation is determined to extract the statistics of relative motion between scatterers. Each Eiger pixel can be treated as an individual detector that measures the intensity at a specific point in space (pixel position) and time (frame number). The intensity autocorrelation, 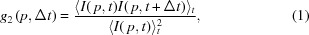
of every pixel was calculated offline, where *I*(*p*,*t*) corresponds to the intensity in pixel (*p*) at time (*t*). Important groundwork for this type of correlation analysis on data from two-dimensional detectors has been published by Sandy *et al.* (1999 ▶), Lumma *et al.* (2000 ▶) and Falus *et al.* (2004 ▶).

**Figure 5:**
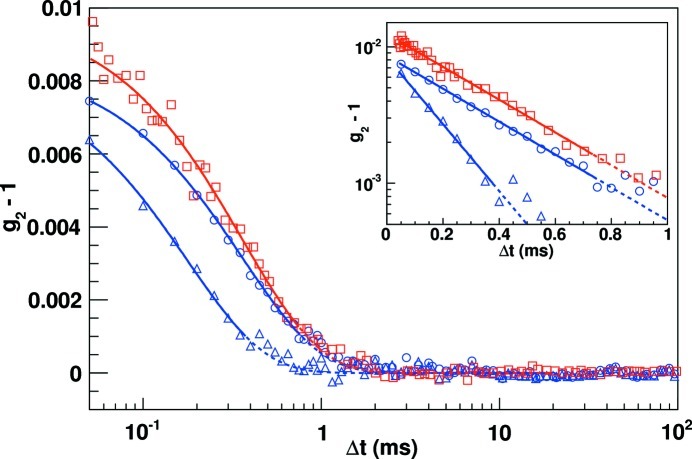
Measured intensity autocorrelation functions. Two measurements at *q* = 0.02957 nm^−1^ of a screened, 1 mm salt concentration, suspension: red boxes, measured with the standard point detector set-up; blue circles, average correlation function from Eiger pixels in a narrow *q* range (d*q* ± 0.001 nm^−1^). One Eiger measurement at higher spatial frequencies *q* = 0.04 nm^−1^, blue triangles, of the weakly screened sample that was extracted from data of 12 exposure series. The curves are pure exponential fits to the initial slopes. The insert shows the slopes and decay parameters on a log(*y*) scale. In the insert the point detector data (red boxes) and curve have been multiplied by 1.25 to vertically offset and visually separate them from the Eiger data (blue circles).

The average correlation function in equilateral *q* rings (0.001 nm^−1^) was computed from an intensity-weighted average of *g*_2_. A combined intensity autocorrelation function for spatial frequencies around *q* = 0.0296 nm^−1^ (∼150 pixels) of one 2.5 s exposure series of 50000 frames is shown in Fig. 5 ▶ (circles). For validation, the autocorrelation was also determined from a classical scintillation point detector (squares in Fig. 5 ▶). In this case the detector size was determined by 75 µm × 75 µm slits directly in front of the point detector to be equivalent to the geometric size of an Eiger pixel. Single-photon signals were amplified, discriminated on both a lower and upper limit, and translated into a TTL pulse train, which was used as input of a multiple-tau hardware correlator, ALV/LSE-5003. In order to achieve comparable statistics, the point-detector autocorrelation functions were derived from a 300 s measurement.

The intensity autocorrelation (1) is related to the field autocorrelation, *g*_1_, by the Siegert relation, 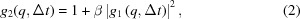
where β is the visibility of the correlation function. *g*_1_ is easily related to the ensemble average of the colloidal conformation and, for short correlation times, decays exponentially according to 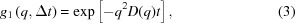
where *D*(*q*) is the short-time diffusion coefficient and 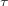 = [*q* ^2^ *D*(*q*)]^−1^ is the decay constant. While it is well known that particle–particle interactions, polydispersity, *etc.* can cause deviations from this simple model (Nägele, 1996 ▶; Sergè & Pusey, 1996 ▶; Holmqvist & Nägele, 2010 ▶) and different short-time and long-time collective diffusion coefficients may result, we find that the data of our demonstration experiment are well described by the single-exponential decay, equation (3).

Fitting the distributions in Fig. 5 ▶ with this model we determined relaxation times τ of 0.722 ± 0.011 ms and 0.724 ± 0.029 ms for the Eiger and point detector data, respectively. The two measurements agree well with each other. While the difference in detection may have an effect on the visibility β, it affects neither the functional form of *g*_1_ nor τ. The visibility depends purely on the optics of the set-up. The slight difference between the two measurements is associated with a small difference in the effective pixel sizes. Furthermore, the importance of smaller pixels is illustrated by the intrinsically low value of β, ∼0.8%, which is a direct reflection of the fairly small speckle size (σ) to pixel size (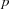) ratio of 0.3 according to σ/*p* = 1/4^1/2^(1/β + 1). This is consistent with an illumination size of less than 20 µm following the equation σ = λ*d*/*a*, where *d* is the sample-to-detector distance and *a* is the size of the illumination. The lower uncertainty of the Eiger data is achieved with the ensemble of the 150 individual *q*-equivalent pixels, *i.e.* many simultaneous but independent measurements.

**Figure 6:**
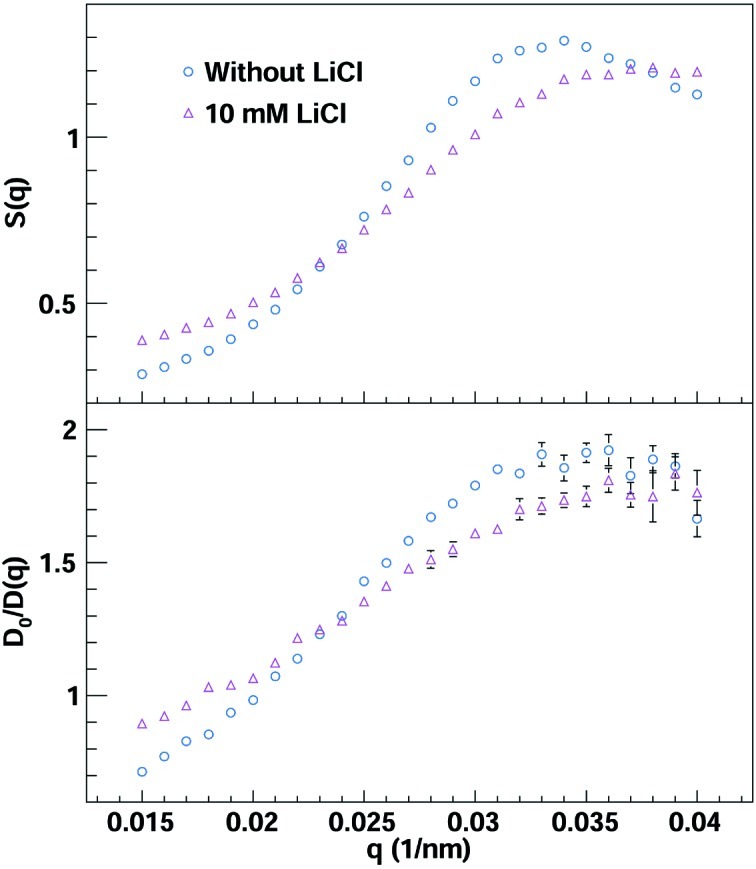
(In colour online.) The structure factors *S*(*q*) and normalized inverse diffusion coefficients *D*_0_/*D*(*q*) for the weakly screened (blue circles) and a screened (pink triangles) suspension.

These measurements demonstrate one clear strength of Eiger, which is the ability to simultaneously capture the scattering angle distributions of the structure factor *S*(*q*) by SAXS and the diffusion coefficients *D*(*q*) *via* XPCS. *S*(*q*) and the normalized inverse diffusion coefficient *D*_0_/*D*(*q*), where *D*_0_ = 2.9 × 10^−8^ cm^2^ s^−1^ is the diffusion coefficient at infinite dilution, are shown in Fig. 6 ▶ for both the weakly screened and a screened suspension. These results have been extracted from 12 exposure series of 2.5 s for a total acquisition time of 30 s, *i.e* 600000 images for each sample, to increase the statistical quality of the results. This statistical increase is especially beneficial at larger *q* values where the combination of lower fluxes and faster decay constants leads to larger uncertainties; an autocorrelation function at *q* = 0.04 nm^−1^ is also shown in Fig. 5 ▶.

## 5. Conclusion

For the first time typical submillisecond relaxation times are now accessible with a large-area pixel detector. Exposure times, and thus radiation damage of the sample, can be greatly reduced with the statistical increase from combining the correlation data of many pixels at similar scattering angles. This substantial statistical gain also increases the sensitivity and ability to measure weakly scattering systems. Furthermore, along with fast intensity auto-correlation measurements, such a pixel detector also allows for the collection of pixel-to-pixel intensity cross correlations (Wochner *et al.*, 2009 ▶). Imaging techniques that require many frames like those proposed in the 1970s (Kam, 1977 ▶) have recently attracted renewed interest owing to the availability of high-brilliance X-ray sources (Saldin *et al.*, 2010 ▶). We anticipate that the smaller pixel size, higher frame rate and negligible readout dead-time of Eiger will enhance these and many other techniques at X-ray synchrotron sources.
